# A Fusion-Based Approach for Breast Ultrasound Image Classification Using Multiple-ROI Texture and Morphological Analyses

**DOI:** 10.1155/2016/6740956

**Published:** 2016-12-29

**Authors:** Mohammad I. Daoud, Tariq M. Bdair, Mahasen Al-Najar, Rami Alazrai

**Affiliations:** ^1^Department of Computer Engineering, German Jordanian University, Amman, Jordan; ^2^Jordan University Hospital, The University of Jordan, Amman, Jordan

## Abstract

Ultrasound imaging is commonly used for breast cancer diagnosis, but accurate interpretation of breast ultrasound (BUS) images is often challenging and operator-dependent. Computer-aided diagnosis (CAD) systems can be employed to provide the radiologists with a second opinion to improve the diagnosis accuracy. In this study, a new CAD system is developed to enable accurate BUS image classification. In particular, an improved texture analysis is introduced, in which the tumor is divided into a set of nonoverlapping regions of interest (ROIs). Each ROI is analyzed using gray-level cooccurrence matrix features and a support vector machine classifier to estimate its tumor class indicator. The tumor class indicators of all ROIs are combined using a voting mechanism to estimate the tumor class. In addition, morphological analysis is employed to classify the tumor. A probabilistic approach is used to fuse the classification results of the multiple-ROI texture analysis and morphological analysis. The proposed approach is applied to classify 110 BUS images that include 64 benign and 46 malignant tumors. The accuracy, specificity, and sensitivity obtained using the proposed approach are 98.2%, 98.4%, and 97.8%, respectively. These results demonstrate that the proposed approach can effectively be used to differentiate benign and malignant tumors.

## 1. Introduction

Breast cancer is the most common cancer in women worldwide and one of the major causes of death in females across the globe [1]. The statistics of the World Health Organization (WHO) indicate that, in 2012, 1.67 million new cases were diagnosed with breast cancer and around 522,000 women died of this disease [1]. Early diagnosis of breast cancer is crucial for the successful treatment of the disease and improving the survival rates of the patients [2].

Ultrasound imaging is one of the most widely used imaging modalities for breast cancer diagnosis since it offers the advantages of low-cost, portability, patient comfort, and diagnosis accuracy [3, 4]. However, the interpretation of breast ultrasound (BUS) images is operator-dependent and varies based on the experience and skill of the radiologist [5]. To overcome this limitation, computer-aided diagnosis (CAD) systems have been introduced to analyze BUS images and provide the radiologist with a second opinion to improve the diagnosis accuracy and reduce the effect of operator dependency [5, 6].

Many studies, such as [7–15], have employed BUS image analysis for classifying breast tumors. In particular, morphological features [13, 16, 17] and texture features [8, 12] are demonstrated to be useful for differentiating benign and malignant tumors. Moreover, combining both feature groups has been suggested to improve the tumor classification accuracy [13, 18]. Morphological features quantify the geometrical characteristics of the tumor, such as area, shape, orientation, regularity, and margins [6, 19]. Therefore, morphological features are mainly affected by the accuracy of the tumor outline. Commonly used morphological feature descriptors include the aspect ratio [13, 17], the best-fit ellipse of the tumor, the normalized radial length (NRL) [18, 20], and the undulation characteristics [21].

Texture features quantify the pixel gray-level statistics in terms of intensity and spatial distribution [6]. Generally, the texture patterns of benign tumors are different from those of malignant tumors [10]. Therefore, several texture descriptors have been employed for classifying BUS images [22–26]. Among these descriptors, the gray-level cooccurrence matrix (GLCM) [27] is one of the most widely used texture analysis techniques for BUS image classification [12]. Conventional texture analysis often uses a single region of interest (ROI) to extract global texture features that quantify the texture characteristics of the entire tumor. One of the most common ROI selection procedures is to find the minimum bounding rectangle that encloses the tumor [9, 12, 22]. Another ROI selection approach is to find the maximum rectangle that fits inside the tumor [28]. Such ROIs can be drawn manually by a radiologist or detected automatically using a computer algorithm.

**Figure 1:**
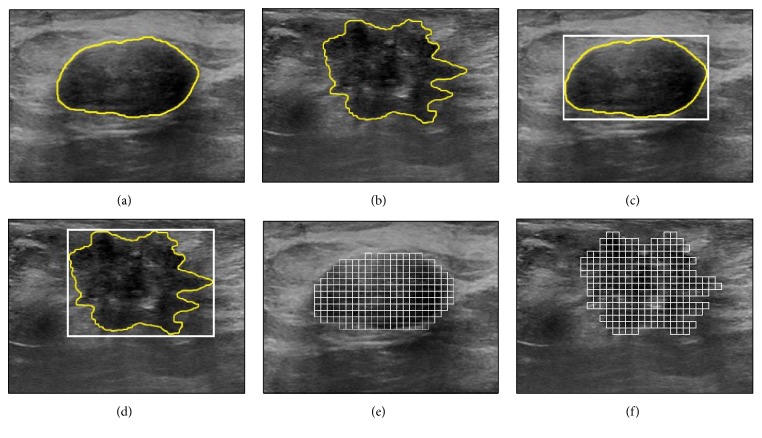
(a)-(b) BUS images of (a) benign and (b) malignant tumors with manually drawn outlines (yellow line). (c)-(d) A single ROI is drawn around each tumor in (a) and (b), such that the ROI corresponds to the minimum bounding rectangle that contains the tumor. Such ROI is often used in conventional texture analysis. (e)-(f) Each tumor in (a) and (b) is divided into a set of nonoverlapping ROIs. These multiple ROIs are used in the proposed approach to extract the texture features.

In many BUS images, the local texture patterns within the tumor vary from one region to another. Hence, the use of a single ROI, which enables the extraction of global texture features that quantify the entire tumor, might not support effective quantification of the local texture variations within the tumor. Moreover, the mismatch between the predefined structure of the ROI and the actual shape of the tumor might reduce the tumor classification accuracy. For example, consider the benign and malignant tumors shown in Figures 1(a) and 1(b), respectively. The texture patterns inside each tumor demonstrate local variations. For both tumors, the ROIs corresponding to the minimum bounding rectangle that encloses the tumor are presented in Figures 1(c) and 1(d). Both ROIs might not provide efficient extraction of texture features that can effectively quantify the local texture variations within the tumor. In addition, the ROI of each tumor extends beyond the tumor boundary, and hence the texture features extracted from such ROI are expected to quantify both the tumor and the surrounding healthy tissue. These limitations might lead to imprecise texture analysis of the tumor, which in turn can reduce the tumor classification accuracy.

To improve the tumor classification capability of ultrasound texture analysis, this study investigates the use of multiple ROIs to analyze the local pixel gray-level statistics inside the tumor. In particular, the tumor is divided into a set of nonoverlapping ROIs as illustrated in Figures 1(e) and 1(f). Each ROI is analyzed individually to extract local texture features. The texture features employed in this study are computed using the GLCM matrix. A local tumor class indicator is estimated for each individual ROI by classifying the texture features of that ROI using a well-trained classifier. The class of the tumor can be determine based on the multiple-ROI texture analysis by employing a majority voting mechanism to integrate the local tumor class indicators of all ROIs inside the tumor. The proposed multiple-ROI texture analysis approach enables effective quantification of the local texture patterns inside the tumor without incorporating texture patterns of the healthy tissue that surrounds the tumor.

One challenge of applying the proposed multiple-ROI texture analysis approach is to enable effective combination between the local texture features, which are extracted for each one of the multiple ROIs inside the tumor, with the morphological features that are computed for the entire tumor. Therefore, a novel probabilistic approach is proposed to fuse the tumor classification indicators obtained using the multiple-ROI texture analysis with the tumor classification indicator computed using morphological analysis of the entire tumor. The morphological analysis employed in this paper is based on set of morphological features introduced in previous studies [13, 17, 18, 20, 21, 29] to quantify the shape and contour of the tumor.

To evaluate the performance of the proposed BUS image classification approach, both the multiple-ROI texture analysis and the fusion-based combination between the multiple-ROI texture analysis and morphological analysis are employed to classify a BUS image database that includes 64 benign tumors and 46 malignant tumors. These BUS images were acquired during ultrasound breast cancer screening procedures. The tumor classifications results of the proposed approach are compared with conventional texture (single ROI), morphological, and combined texture and morphological analyses.

The remainder of the paper is organized as follows. The data acquisition of the BUS image database is summarized in Section 2. Moreover, Section 2 describes the conventional texture and morphological analysis of BUS images, the proposed tumor classification approach, and the performance metrics employed to compare the conventional and proposed BUS image classification approaches. The experimental results and discussion are provided in Section 3. Finally, the conclusion is presented in Section 4.

## 2. Materials and Methods

### 2.1. Data Acquisition

The collected image database consists of 110 BUS images of pathologically proven benign and malignant tumors (64 benign tumors and 46 malignant tumors). Detailed description of the types of benign and malignant tumors involved in this study is provided in Table 1. Each BUS image was acquired from one patient (i.e., the number of patients which participated in the study is 110). All participated patients were females. Moreover, each image included exactly one breast tumor. The age of the patients ranged from 25 to 77 years. The mean and standard deviation of the maximum diameters of the tumors are 14.7 mm and 6.0 mm, respectively. The BUS images were acquired during routine ultrasound breast cancer screening procedures at the Jordan University Hospital, Amman, Jordan, during the period between May 2012 and February 2016. Ultrasound imaging was performed using an Acuson S2000 ultrasound system (Siemens AG, Munich, Germany) and a 14L5 linear transducer with frequency bandwidth from 5 to 14 MHz. During imaging, the radiologist was free to adjust the configurations of the imaging system, including the focal length, depth, and gain to obtain the best view. For each BUS image, the tumor was manually outlined by a radiologist with more than 13 years of experience. The tumor outlines were also verified by another independent experienced radiologist. All images were resampled to the same resolution of 0.1 mm × 0.1 mm per pixel. The study protocol was approved by the ethics committee at the Jordan University Hospital. Moreover, informed consent to the protocol was obtained from each patient.

### 2.2. Quantitative Features

Both texture and morphological features are used to classify the benign and malignant breast tumors. The following two sections describe both feature groups.

#### 2.2.1. Texture Features

The texture features employed in this study were computed using the GLCM matrix [27], which measures the correlations between adjacent pixels within a ROI. The computation of the GLCM matrix was performed using four distances (*d* = 1, 2, 3, and 4 pixels) and four different orientations (*θ* = 0°, 45°, 90°, and 135°). Therefore, sixteen GLCM matrices were computed for each ROI. Each GLCM matrix was analyzed, as described in [12], to extract twenty texture features (TF1–TF20) that are commonly used for ultrasound texture analysis [12, 32]. These texture features are provided in Table 2. Thus, a total of 320 texture features were extracted from each ROI.

#### 2.2.2. Morphological Features

In this study, eighteen morphological features are extracted from each tumor. Among these features, ten features can be extracted directly from the tumor (MF1–MF10). Six morphological features are extracted from the best-fit ellipse that approximates the size and position of the tumor (MF11–MF16). The last two morphological features are the entropy (MF17) and variance (MF18) of the normalized radial length (NRL) of the tumor [18, 20]. The NRL is defined as the distance between the tumor center and the pixels located on the tumor boundary normalized to the maximum radial length of the tumor [18]. The eighteen morphological features are summarized in Table 2.

### 2.3. Conventional Tumor Classification

**Figure 2:**
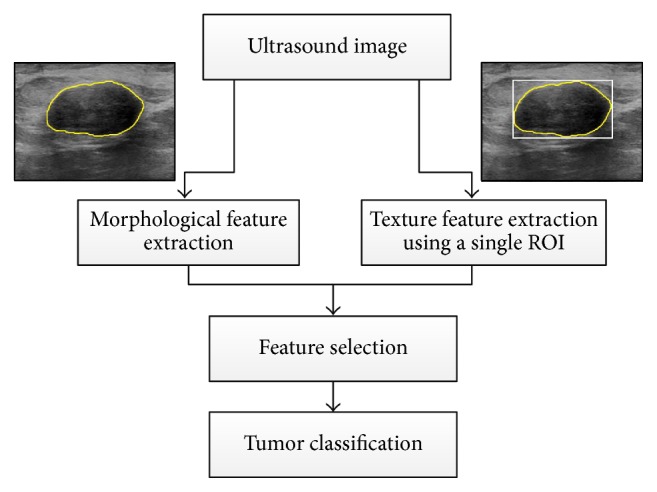
Overview of conventional tumor classification in which texture features are extracted from a single ROI that encloses the tumor and morphological features are computed based on the tumor outline. Both groups of features are processed using feature selection and classification to differentiate benign and malignant tumors.

The 110 BUS images are analyzed using conventional tumor classification analysis, as illustrated in Figure 2. In particular, the GLCM texture features, described in Section 2.2.1, are extracted from a single ROI. As mentioned in the Introduction, this ROI corresponds to the minimum bounding rectangle that encloses the tumor. The morphological features, summarized in Section 2.2.2, are extracted from the outlined tumor.

Feature selection, which eliminates the irrelevant and redundant features, is applied to determine the best subsets of texture, morphological, and combined texture and morphological features that reduce the misclassification error between malignant and benign tumors. In fact, exhaustive search for the optimal feature combination requires extensive computational resources and long processing times, particularly when the number of features is large. For example, the total number of all potential combinations of *n* features into *m* subsets is equal to $\left(1/m!\right)\sum_{i=0}^{m}{\left(-1\right)}^{m-i}\left(\begin{array}{c} m\\ i \end{array}\right)i^{n}$ [33]. Therefore, a two-phase heuristic approach, which is based on the feature selection procedures described in [12, 34], is employed to carry out feature selection. In the first phase, the features are ranked according to the minimal-redundancy-maximal-relevance (mRMR) criterion [34], which is based on mutual information. The top* l*-ranked features are incrementally grouped and their classification performance is evaluated, for all *l* = {1, 2, …, *L*}, where *L* is the total number of features. The smallest feature group that can achieve the minimum classification error is taken as the candidate feature subset. In the second phase, the backward selection algorithm is applied to the candidate feature subset. In this algorithm, features are sequentially eliminated until the removal of further features leads to degrading the classification accuracy. This two-phase algorithm enables the selection of a compact feature subset that can achieve effective tumor classification.

The selected features are classified using a binary SVM classifier [35] that is implemented using the LIBSVM library [36]. In binary SVM, the input features are mapped into a high dimensional feature space by applying a kernel function. This mapping enables the computation of a nonlinear decision function that can separate the feature space into two regions, one for each class. Specifically, given a training set *T* = {(**x**_1_, *y*_1_),…, (**x**_*k*_, *y*_*k*_),…, (**x**_*n*_, *y*_*n*_)}, where **x**_*k*_ ∈ *R*^*N*^ represents the* k*th feature vector and *y*_*k*_ ∈ {−1, +1} is the corresponding tumor class. The goal of SVM is to determine a decision boundary in the form of hyperplane that can separate the feature space into two regions through maximizing the margin between the samples of different classes. The resultant decision function is defined as follows:

(1) $$\begin{array}{c} f\left(\;x\;\right)=sgn\left(\;\overset{n}{\underset{k=1}{\sum }}y_{k}\alpha_{k}\varphi \left(\;x_{k},x\;\right)+b\;\right), \end{array}$$

where **x** ∈ *R*^*N*^ is a new feature vector to be classified into benign or malignant, *φ*(**x**_*k*_, **x**) is a kernel function that maps the input vectors into high dimensional space, *α*_*k*_ is the* k*th Lagrange multiplier, and *b* is the bias term of the decision hyperplane. Several kernel functions can be used with SVM. However, the Gaussian radial basis function (RBF) is by far the most commonly used kernel function for classification tasks [37]. In this work, the RBF kernel is employed. The RBF kernel function can be defined as follows:

(2) $$\begin{array}{c} \varphi \left(\;x_{k},x\;\right)=\exp\left(\;-\frac{{\left\|x_{k}-x\right\|}^{2}}{2\sigma^{2}}\;\right), \end{array}$$

where *σ* > 0 is the RBF kernel parameter.

The performance of the SVM classifier with RBF kernel depends on two parameters: *σ*, the RBF kernel parameter, and *C* > 0, the regularization parameter. The tuning of the two parameters is carried out using a grid-based search of the two-dimensional parameter space 1 < *σ* < 100 and 1 < *C* < 100. The search is performed with a step length of 1. The best SVM model is selected such that its parameters maximize the average tumor classification accuracy.

The performance evaluation of the conventional tumor classification is performed using the single ROI GLCM texture features, the morphological features, and the combined single ROI texture features and morphological features. Similar to the work of Wu et al. [13], the evaluation is carried out using a fivefold cross-validation procedure. In this procedure, 80% of the tumors are selected for training and the remaining 20% is used for testing. This process is repeated five times so that each of the 110 BUS images is included once in the testing.

### 2.4. The Proposed Tumor Classification Approach

**Figure 3:**
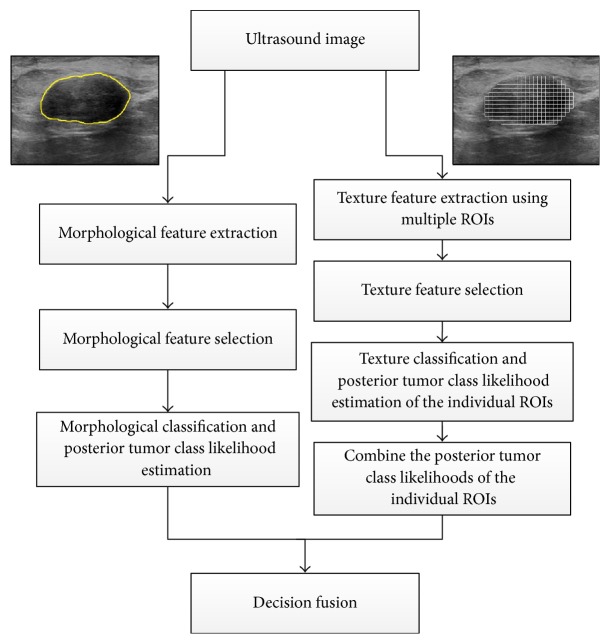
The architecture of the proposed tumor classification approach. Texture analysis is performed by dividing the tumor into a group of nonoverlapping ROIs and extracting texture features from each ROI. A selected set of texture features are used to classify each individual ROI and compute its posterior tumor class likelihood. The posterior tumor class likelihoods of the individual ROIs are combined. Morphological analysis is performed by extracting morphological features from the outlined tumor and employing a selected set of the features to predict the posterior tumor class likelihood. Decision fusion is then used to combine the posterior tumor class likelihoods obtained using the texture and morphological analyses and determine the tumor class.

The architecture of the proposed tumor classification approach is illustrated in Figure 3. In this architecture, the multiple-ROI texture analysis is carried out by dividing the tumor into small, nonoverlapping ROIs and extracting local texture features from each individual ROI. Moreover, the tumor is analyzed to extract morphological features. To combine the local texture features of the individual ROIs and the global morphological features, two independent posterior tumor class likelihoods are obtained separately from the multiple-ROI texture analysis and the morphological analysis. Moreover, decision fusion is applied to fuse both tumor class likelihoods and determine the class of the tumor.

**Figure 4:**
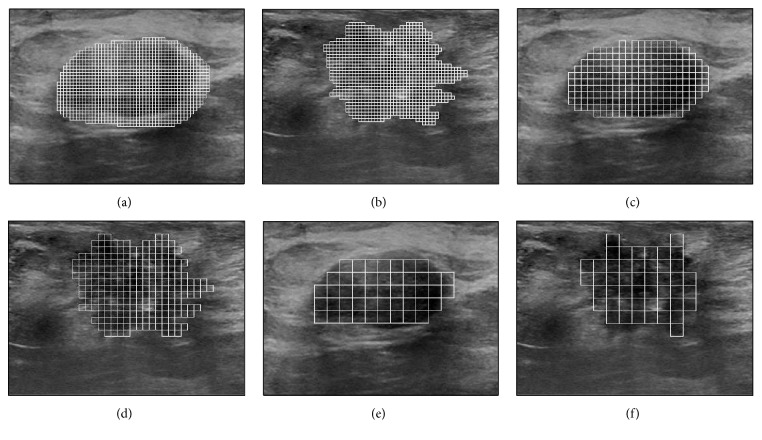
The benign and malignant tumors shown in Figures 1(a) and 1(b), respectively, are divided into a set of nonoverlapping ROIs with a size of (a)-(b) 0.5 × 0.5 mm^2^, (c)-(d) 1 × 1 mm^2^, and (e)-(f) 2 × 2 mm^2^.

To perform the multiple-ROI texture analysis, the tumor is divided into a set of uniform, nonoverlapping ROIs, as shown in Figures 1(e) and 1(f). The size of the ROIs is estimated by considering three factors: preserving the capability of differentiating various texture patterns, reducing the possibility of including different local textures within the same ROI, and ensuring that the entire tumor is adequately covered by the ROIs. The study by Valckx and Thijssen [38] suggested that the use of very small ROIs might degrade the capability of differentiating various texture patterns. On the other hand, the use of large ROIs increases the possibility of including different local texture patterns within a single ROI. Moreover, the use of large ROIs might lead to big gaps: that is, areas that are not covered by the ROIs, at the tumor boundary. For example, consider Figures 4(a), 4(c), and 4(e) that show the benign tumor in Figure 1(a) divided into uniform ROIs of size 0.5 × 0.5 mm^2^, 1 × 1 mm^2^, and 2 × 2 mm^2^, respectively. Moreover, consider Figures 4(b), 4(d), and 4(f) that show the malignant tumor in Figure 1(b) divided into ROIs of sizes 0.5 × 0.5 mm^2^, 1 × 1 mm^2^, and 2 × 2 mm^2^, respectively. The use of the 0.5 × 0.5 mm^2^ ROIs minimizes the possibility of including different local textures within a single ROI and reduces the gaps at the tumor boundary. However, the small size of the ROIs, which corresponds to 5 × 5 pixels, might limit the ability of the texture analysis to differentiate various texture patters. On the other hand, the use of the 2 × 2 mm^2^ ROIs, which correspond to 20 × 20 pixels, enables better texture classification but increases the possibilities of including different local textures within the same ROI and producing large gaps at the tumor boundary. The 1 × 1 mm^2^ ROIs, which correspond to 10 × 10 pixels, provide a reasonable balance between the need to use ROIs of reasonable size to enable effective texture analysis and the requirements of reducing the possibility of crossing different local textures within a single ROI and achieving adequate coverage of the entire tumor. Hence, the size of the ROIs employed in this study is set to 1 × 1 mm^2^.

Each ROI is processed individually to extract the GLCM texture features described in Section 2.2.1. The two-phase feature selection algorithm described in Section 2.3 is employed to determine the subset of texture features that enables the best tumor classification accuracy based on the multiple-ROI texture analysis. A binary SVM classifier with RBF kernel is used to classify each ROI as benign or malignant using the selected subset of texture features. The tuning of the SVM parameters is achieved using the grid-based search described in Section 2.3. The posterior tumor class likelihood of each ROI is estimated from the SVM output using Platt's approach [39]. Then, a majority voting mechanism is used to determine the class of the tumor based on the classification indictors of the individual ROIs. In particular, if more than 50% of the ROIs in the tumor are classified as malignant, then the tumor is considered malignant. Otherwise, the tumor is considered benign. The computation of the posterior likelihood of the tumor is performed by averaging the posterior tumor class likelihoods of the ROIs that agree with the class of tumor estimated using the multiple-ROI texture analysis.

To perform the morphological analysis, the extraction and selection of the morphological features as well as the tuning of the SVM classifier match those of the conventional morphological-based classification that was described in Section 2.3. Moreover, the tuned SVM is used to classify the tumor based on the selected morphological features and Platt's approach is applied to compute the posterior tumor class likelihood of the entire tumor.

For a given BUS image, the posterior tumor class likelihood obtained using the multiple-ROI texture analysis is mutually independent from the posterior tumor class likelihood estimated using the morphological analysis. Therefore, the fusion of the tumor class decisions obtained using these two independent analyses can be performed using a Gaussian Naive-Bayes approach [40].

To apply the Gaussian Naive-Bayes approach, consider a vector of continuous decisions **D** = [*d*_1_,…,*d*_*L*_]^*T*^ obtained from *L* different classifiers for a specific BUS image. The probability that the BUS image belongs to class *y* given decisions of the *L* different classifiers can be written as

(3) $$\begin{array}{c} P\left(\;y\;|\;d_{1},\ldots ,d_{L}\;\right)=\frac{P\left(y\right)P\left(d_{1},\ldots ,d_{L}\;|\;y\right)}{P\left(d_{1},\ldots ,d_{L}\right)}, \end{array}$$

where for binary classification, which is considered in this study, *y* ∈ {−1,1} and *L* = 2. Using the mutual independence assumption between the two classifiers, (3) can be rewritten as

(4) $$\begin{array}{c} P\left(\;y\;|\;d_{1},\ldots ,d_{L}\;\right)=\frac{P\left(y\right)\prod_{i=1}^{L}P\left(d_{i}\;|\;y\right)}{P\left(d_{1},\ldots ,d_{L}\right)}. \end{array}$$

The term *P*(*d*_1_,…, *d*_*L*_) is a normalization factor. Therefore, a BUS image can be classified based on the combined decisions from the *L* = 2 classifiers using the following decision rule:

(5) $$\begin{array}{c} \hat{y}=\underset{y}{\arg\;\max}\left(\;P\left(\;y\;\right)\overset{L}{\underset{i=1}{\prod }}P\left(\;d_{i}\;|\;y\;\right)\;\right), \end{array}$$

where *P*(*d*_*i*_∣*y*) is assumed to be a multivariant normal distribution with mean vector *μ*_*i*_ and covariance matrix *C*_*i*_ ∈ *R*^*L*×*L*^. The class prior probability *P*(*y*) and the parameters (*μ*_*i*_, *C*_*i*_) are estimated using maximum likelihood [41].

The performance evaluation of the proposed tumor classification approach is carried out using two different configurations. In the first configuration, the tumor is classified using the multiple-ROI texture analysis only. In the second configuration, tumor classification is carried out by fusing the posterior tumor class likelihoods of the multiple-ROI texture analysis and the morphological analysis. In both configurations, the fivefold cross-validation procedure described in Section 2.3 is employed. It is worth noting that the selection of the ROIs during the fivefold SVM training and testing of the multiple-ROI texture analysis was tumor-specific. In other words, in each fold of the cross-validation procedure, the training was performed using ROIs that belong to 80% of the tumors, while the testing was carried out with the ROIs of the remaining 20% of the tumors.

### 2.5. Performance Evaluation

Six objective metrics, namely, the accuracy, specificity, sensitivity, negative predictive value (NPV), positive predictive value (PPV), and Matthew's correlation coefficient (MCC) [6], are used to evaluate the performance of the conventional tumor classification as well as the proposed tumor classification. These metrics are defined as follows:

(6) Accuracy=TP+TNTP+TN+FP+FN,Specificity=TNTN+FP,Sensitivity=TPTP+FN,PPV=TPTP+FP,NPV=TNTN+FN,MCC=TP×TN−FP×FNTP+FPTP+FNTN+FPTN+FN,

where TP is the number of true positive cases, TN is the number of true negative cases, FP is the number of false positive cases, and FN is the number of false negative cases.

The relationships between specificity and sensitivity, achieved using the conventional and proposed classification approaches, are analyzed by drawing the receiver operator characteristic (ROC) curves. Moreover, the area under the ROC curve (AUC), which quantifies the overall performance of a CAD system, is computed for each classification approach.

To confirm the effectiveness of the proposed fusion-based approach, paired *t* tests were carried out on average classification accuracies to compare the fused multiple-ROI texture and morphological analyses with the other four classification approaches.

The execution times of the conventional texture, morphological, and combined texture and morphological analyses are compared with the proposed multiple-ROI texture analysis and the fused multiple-ROI texture and morphological analyses. The compression was performed by implementing the five approaches using MATLAB (MathWorks Inc., Natick, Massachusetts, USA) and executing them on a computer workstation that has a 3.5 GHz processor and 16 GB of memory and runs Ubuntu Linux operating system. For each one of the five classification approaches, the total time required to extract the features and classify the BUS image was recorded for twenty trials.

## 3. Results and Discussion

The tuned values of the SVM parameters (*σ*, *C*) that are used to carry out tumor classification using the conventional texture features, morphological features, and combined texture and morphological features are equal to (3,56), (3,50), and (2,50), respectively. Moreover, the tuned values of (*σ*, *C*) that are employed to perform tumor classification using the proposed multiple-ROI texture analysis are equal to (4,55). To carry out the fusion-based tumor classification, both the multiple-ROI texture analysis and the morphological analysis are performed using their optimized SVM parameters (i.e., the parameters (4,55) are used for the multiple-ROI texture analysis and (3,50) are employed for the morphological analysis).

The features selected to perform the proposed multiple-ROI texture analysis are TF1_4,90°_, TF2_3,90°_, TF4_4,90°_, TF6_4, 90°_, TF8_4,90°_, TF9_4, 90°_, TF10_3,90°_, TF11_3,45°_, TF12_3,45°_, TF13_3,135°_, TF14_4,45°_, TF15_2,90°_, TF16_2,90°_, TF17_4,90°_, and TF18_4,90°_, where the first subscript is the distance,* d*, and the second is the orientation angle,*θ*. The proposed fusion-based tumor classification was performed using the aforementioned multiple-ROI texture features as well as the selected subset of morphological features. These morphological features are MF1, MF2, MF3, MF4, MF5, MF6, MF7, MF8, MF13, MF14, and MF18.

The results achieved by the proposed tumor classification approach using the multiple-ROI texture analysis as well as the fused multiple-ROI texture and morphological analyses are shown in Table 3 with respect to the pathological findings. Both configurations of the proposed approach achieved effective classification of benign and malignant breast tumors. However, the fusion of the multiple-ROI texture analysis and morphological analysis enabled higher classification performance than that obtained using the multiple-ROI texture analysis alone.

The six objective performance metrics obtained for the proposed classification approach and conventional classification approach are presented in Table 4. The conventional classification approach achieved better performance by combining the texture and morphological features than that obtained by only using the texture features or the morphological features. This finding agrees with the results reported in previous studies [13, 14]. Moreover, the classification results demonstrate that the proposed approach using the multiple-ROI texture analysis outperforms the conventional classification using the texture, morphological, and combined texture and morphological features. In particular, the multiple-ROI texture analysis achieved classification accuracy of 95.5%, specificity of 93.8%, sensitivity of 97.8%, PPV of 91.8%, NPV of 98.4%, and MCC of 90.9%. The optimal classification performance was achieved by the proposed approach using the fused multiple-ROI texture analysis and morphological analysis. Specifically, the fusion of the multiple-ROI texture and morphological analyses enabled classification accuracy of 98.2%, specificity of 98.4%, sensitivity of 97.8%, PPV of 97.8%, NPV of 98.4%, and MCC of 96.3%.

**Figure 5:**
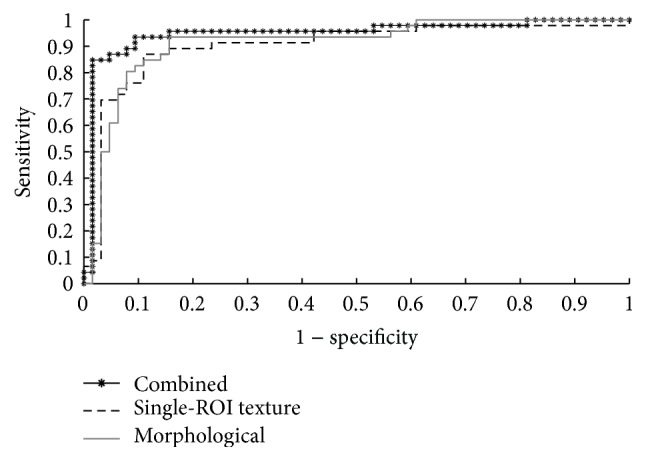
The ROC curves of the conventional classification approach using texture features, morphological features, and the combined texture and morphological features.

**Figure 6:**
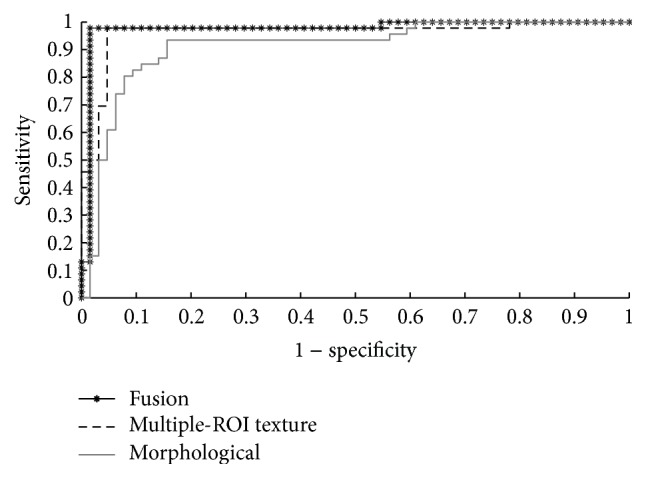
The ROC curves of the proposed classification approach using the multiple-ROI texture analysis, the morphological analysis, and the fused multiple-ROI texture analysis and morphological analysis.

The ROC curves of the conventional classification approach and the proposed classification approach are shown in Figures 5 and 6, respectively. The AUC values obtained for the conventional classification using the texture features, morphological features, and combined texture and morphological features are equal to 0.902, 0.912, and 0.948, respectively. The proposed classification approach achieved AUC values of 0.963 using the multiple-ROI texture analysis and 0.975 using the fused multiple-ROI texture and morphological analyses. These results confirm the superior performance of the proposed classification approach compared to conventional BUS image classification.

The *p* values obtained using the paired *t* tests to compare the proposed fused multiple-ROI texture and morphological analyses with the other four classification approaches at confidence level of 0.05 are shown in Table 5. The results reported in Table 5 demonstrate that the fusion-based approach outperforms significantly the conventional classification using the texture features, morphological features, and combined texture and morphological features as well as the multiple-ROI texture analysis.

According to these results, our proposed tumor classification approach achieved high sensitivity of 97.8% using both the multiple-ROI texture analysis and the fused multiple-ROI texture and morphological analyses. Such finding suggests that the proposed approach enables high probability of diagnosing malignant tumors. Moreover, the near-perfect values of PPV and NPV obtained by fusing the multiple-ROI texture analysis and morphological analysis indicate that the number of unnecessary biopsies carried out for benign tumors can be minimized. These results also suggest that the proposed approach has the potential to provide the radiologists with a second opinion that effectively reduces the rate of misdiagnosis.

The mean ± standard deviation execution times of the multiple-ROI texture analysis and the fused multiple-ROI texture and morphological analyses are 72.20 ± 2.14 s and 73.66 s ± 2.19 s, respectively. In comparison, the mean ± standard deviation execution times of the conventional texture, morphological, and combined texture and morphological analyses are 0.16 ± 0.03 s, 1.47 ± 0.18 s, and 1.63 ± 0.19 s, respectively. Although the multiple-ROI texture analysis and the fused multiple-ROI texture and morphological analyses are slower than the conventional classification analyses, both proposed classification approaches require around one minute to classify the BUS image. Such execution times do not limit the application of the proposed classification approaches in CAD systems that aim to provide an accurate second opinion to the radiologist.

The results reported in this study indicate that the proposed multiple-ROI texture analysis outperforms the conventional texture analysis in which texture features are extracted from a single ROI that includes the tumor. As mentioned in the Introduction, many breast tumors might have complicated texture patterns that vary from one region to another inside the tumor. Therefore, the multiple-ROI texture analysis enables effective quantification of the different local texture patterns inside the tumor. Another factor that might contribute to the improved performance of the multiple-ROI texture analysis is its ability to analyze the local texture patterns of the tumor without incorporating texture patterns of the surrounding healthy tissue.

The use of small ROIs for tissue characterization has been employed by other ultrasound-based methods. For example, in quantitative ultrasound imaging of cancer [42, 43], the raw ultrasound radio-frequency (RF) signals are divided into small ROIs, and each ROI is analyzed to extract spectral features for tissue characterization. Moreover, a recent study by Uniyal et al. [44] has compared the classification performance of a combination of ultrasound-based texture, spectral, and RF time series features that are extracted from the entire breast tumor with the performance obtained by dividing the tumor into 1 × 1 mm^2^ ROIs and extracting similar ultrasound-based features from each individual ROI. This study demonstrates that the classification performance obtained by classifying the individual 1 × 1 mm^2^ ROIs outperforms the classification results achieved by classifying the entire tumor. This finding agrees with our proposed multiple-ROI texture analysis approach.

The multiple-ROI texture analysis has been applied in the current study to improve the classification performance of GLCM texture features. Our future directions include extending the multiple-ROI texture analysis approach to incorporate other statistical texture methods that use a ROI to extract texture features. The proposed approach can also be extended by performing multiresolution texture feature extraction, in which ROIs of different sizes are employed to carry out the multiple-ROI texture analysis. Moreover, the probabilistic approach, which has been used in this study to fuse the multiple-ROI texture analysis with the morphological analysis, can be expanded to support the fusion of multiple classification results obtained using various texture and morphological methods with the goal of achieving higher accuracy, specificity, and sensitivity.

One important factor that affects the tumor classification performance is the ability to accurately outline the tumor. In particular, imprecise outlining of the tumor might influence the morphological features that quantify the shape and contour of the tumor. Moreover, the texture features, which are extracted from the outlined tumor region, might also be affected by tumor segmentation errors. In this study, tumor outlining was performed by a radiologist with more than thirteen years of experience. Such manual outlining by an experienced operator has been employed in several previous studies, such as [10, 15]. In fact, the manual outlining of the tumor is a time consuming task and its accuracy is subject to the experience level of the radiologist. The future direction of this work is to employ automatic tumor segmentation algorithms, such as [45], that employ advanced image processing techniques to achieve accurate and objective outlining of the tumors.

The multiple-ROI texture analysis approach employed in this study can be extended to reduce the effect of tumor outlining errors. In particular, for each ROI inside the computer-drawn outline, a well-trained classifier can be used to estimate the probability of belonging to the tumor or the surrounding healthy tissue. Such probability estimation can be used to weight the tumor class indicators obtained from the individual ROIs. A customized voting algorithm can be developed to combine the weighted tumor class indicators of the individual ROIs and estimate posterior tumor class likelihood.

## 4. Conclusion

In this study, an effective approach for BUS image classification is proposed. Texture analysis is carried out by dividing the tumor into a set of nonoverlapping ROIs and processing each ROI individually to estimate its tumor class indicator. The tumor class indicators of all ROIs inside the tumor are combined using a majority voting mechanism to estimate the posterior tumor class likelihood. In addition to the multiple-ROI texture analysis, morphological analysis is used to estimate the posterior tumor class likelihood. A probabilistic approach is employed to fuse the posterior tumor class likelihoods obtained using the texture and morphological analyses. The proposed approach has been employed to classify 110 BUS images. The classification results indicate that the proposed approach achieved classification performance that outperforms conventional texture and morphological analyses. In particular, fusing the multiple-ROI texture analysis and morphological analysis enabled classification accuracy of 98.2%, specificity of 98.4%, and sensitivity of 97.8%. These results suggest that the proposed approach has the potential to provide the radiologists with an accurate second opinion to reduce the rate of expendable biopsy and minimize BUS image misdiagnosis.

## Figures and Tables

**Table 1:** Description of the benign and malignant breast tumors involved in the study.

Tumor class	Description	Number of patients
Benign	Fibroadenoma	35
	Complex fibroadenoma	1
	Fibrocystic change	15
	Chronic inflammation	1
	Lymphocytic lobulitis	1
	Fibrosis	3
	Sclerosing adenosis	1
	Atypical ductal hyperplasia	1
	Atypical lobular hyperplasia	1
	Adenosis	2
	Chronic mastitis	1
	Tubular adenoma	1
	Fat necrosis	1

Malignant	Invasive ductal carcinoma	41
	Ductal carcinoma in situ	4
	Invasive lobular carcinoma	1

**Table 2:** The morphological and texture features employed for tumor classification.

Category	Feature	Code	Description
Texture	Autocorrelation [30]	TF1	Twenty texture features (TF1–TF20) are extracted from GLCM matrices computed using four distances (*d* = 1,2, 3,4 pixels) and four orientations (*θ* = 0°, 45°, 90°, 135°)
	Contrast [12]	TF2	
	Correlation [30]	TF3	
	Cluster prominence [30]	TF4	
	Cluster shade [30]	TF5	
	Dissimilarity [30]	TF6	
	Energy [30]	TF7	
	Entropy [30]	TF8	
	Homogeneity [30]	TF9	
	Maximum probability [30]	TF10	
	Sum of squares [27]	TF11	
	Sum average [27]	TF12	
	Sum entropy [27]	TF13	
	Sum variance [27]	TF14	
	Difference variance [27]	TF15	
	Difference entropy [27]	TF16	
	Information measure of correlation I [27]	TF17	
	Information measure of correlation II [27]	TF18	
	Inverse difference normalized [31]	TF19	
	Inverse difference moment normalized [31]	TF20	

Morphological	Tumor area [20]	MF1	Ten morphological features (MF1–MF10) are extracted directly from the tumor
	Perimeter [20]	MF2	
	Form factor [13, 17]	MF3	
	Roundness [13, 17]	MF4	
	Aspect ratio [13, 17]	MF5	
	Convexity [13, 17]	MF6	
	Solidity [13, 17]	MF7	
	Extent [13, 17]	MF8	
	Undulation characteristics [21]	MF9	
	Compactness [20, 29]	MF10	

Morphological	Length of the ellipse major axis [20]	MF11	Six morphological features (MF11–MF16) are extracted from the best-fit ellipse that approximates the size and position of the tumor
	Length of the ellipse minor axis [20]	MF12	
	Ratio between the ellipse major and minor axes [20]	MF13	
	Ratio of the ellipse perimeter and the tumor perimeter [20]	MF14	
	Overlap between the ellipse and the tumor [20]	MF15	
	Angle of the ellipse major axis [20]	MF16	

Morphological	NRL entropy [18, 20]	MF17	Two morphological features (MF17-MF18) are extracted from the NRL of the tumor
	NRL variance [18, 20]	MF18	

**Table 3:** Classification results of the 110 BUS images obtained using the proposed approach.

BUS image classification	Multiple-ROI texture analysis		Fusion of the multiple-ROI texture analysis and the morphological analysis	
	Benign^*∗*^	Malignant^*∗*^	Benign^*∗*^	Malignant^*∗*^
Benign	60 TN	1 FN	63 TN	1 FN
Malignant	4 FP	45 TP	1 FP	45 TP
Total	64	46	64	46

^*∗*^Histological finding.

**Table 4:** Objective performance metrics obtained using the (a) conventional classification approach using texture features, (b) conventional classification approach using morphological features, (c) conventional classification approach using both texture and morphological features, (d) proposed classification approach using multiple-ROI texture analysis, and (e) proposed classification approach using the fused multiple-ROI texture analysis and morphological analysis.

	(a)	(b)	(c)	(d)	(e)
Accuracy	85.5%	87.3%	90.9%	95.5%	98.2%
Specificity	84.4%	89.1%	90.6%	93.8%	98.4%
Sensitivity	87.0%	84.8%	91.3%	97.8%	97.8%
PPV	80.0%	84.8%	87.5%	91.8%	97.8%
NPV	90.0%	89.1%	93.6%	98.4%	98.4%
MCC	70.7%	73.9%	81.5%	90.9%	96.3%

**Table 5:** Comparisons of the *p* values computed using paired *t*-tests on average accuracies between the fused multiple-ROI texture and morphological analyses and the (a) conventional classification approach using texture features, (b) conventional classification approach using morphological features, (c) conventional classification approach using both texture and morphological features, and (d) multiple-ROI texture analysis.

	(a)	(b)	(c)	(d)
*p* value	0.007	0.011	0.041	0.046
